# Modeling of Cancer Stem Cell State Transitions Predicts Therapeutic Response

**DOI:** 10.1371/journal.pone.0135797

**Published:** 2015-09-23

**Authors:** Mary E. Sehl, Miki Shimada, Alfonso Landeros, Kenneth Lange, Max S. Wicha

**Affiliations:** 1 Division of Hematology-Oncology, Department of Medicine, David Geffen School of Medicine, University of California, Los Angeles, Los Angeles, CA, United States of America; 2 Department of Biomathematics, David Geffen School of Medicine, University of California, Los Angeles, Los Angeles, CA, United States of America; 3 Waseda University, Shinjuku, Japan; 4 Department of Human Genetics, David Geffen School of Medicine, University of California, Los Angeles, Los Angeles, CA, United States of America; 5 Department of Statistics, University of California, Los Angeles, Los Angeles, CA, United States of America; 6 University of Michigan Comprehensive Cancer Center, Ann Arbor, MI, United States of America; Swedish Neuroscience Institute, UNITED STATES

## Abstract

Cancer stem cells (CSCs) possess capacity to both self-renew and generate all cells within a tumor, and are thought to drive tumor recurrence. Targeting the stem cell niche to eradicate CSCs represents an important area of therapeutic development. The complex nature of many interacting elements of the stem cell niche, including both intracellular signals and microenvironmental growth factors and cytokines, creates a challenge in choosing which elements to target, alone or in combination. Stochastic stimulation techniques allow for the careful study of complex systems in biology and medicine and are ideal for the investigation of strategies aimed at CSC eradication. We present a mathematical model of the breast cancer stem cell (BCSC) niche to predict population dynamics during carcinogenesis and in response to treatment. Using data from cell line and mouse xenograft experiments, we estimate rates of interconversion between mesenchymal and epithelial states in BCSCs and find that EMT/MET transitions occur frequently. We examine bulk tumor growth dynamics in response to alterations in the rate of symmetric self-renewal of BCSCs and find that small changes in BCSC behavior can give rise to the Gompertzian growth pattern observed in breast tumors. Finally, we examine stochastic reaction kinetic simulations in which elements of the breast cancer stem cell niche are inhibited individually and in combination. We find that slowing self-renewal and disrupting the positive feedback loop between IL-6, Stat3 activation, and NF-*κ*B signaling by simultaneous inhibition of IL-6 and HER2 is the most effective combination to eliminate both mesenchymal and epithelial populations of BCSCs. Predictions from our model and simulations show excellent agreement with experimental data showing the efficacy of combined HER2 and Il-6 blockade in reducing BCSC populations. Our findings will be directly examined in a planned clinical trial of combined HER2 and IL-6 targeted therapy in HER2-positive breast cancer.

## Introduction

Breast cancer is the most common type of cancer in women, with over 230,000 new cases diagnosed and nearly 40,000 deaths in the United States every year [1]. The majority of deaths are caused by distant recurrence [1]. Stem cell-targeted therapies offer new hope in eradicating breast cancer stem-like cells that lead to recurrence after standard therapies fail [2–4]. Mathematical models have proven useful in studying the population dynamics of cancer stem cells under targeted therapy [5, 6]. Models are also informative in assessing safety and duration of therapy [7]. However, the complexities of the stem cell microenvironment limit the predictive ability of analytic models and suggest the necessity of more detailed models pursued through simulation.

Stochastic reaction kinetics allows simulation of complex biological systems with inherent stochasticity and multiple overlapping feedback and feedforward loops [8, 9]. The goal of stochastic simulation in medicine is to integrate knowledge of biological complexity, to unravel the functional properties of organisms in health, and to interface models of disease states with treatments that might restore health [10–14]. In stochastic simulation, a set of reactant species proceeds through a specified set of reactions. Molecular populations are whole numbers that change by discrete amounts. Each reaction occurs with a reaction propensity that is proportional to the reaction rate constant and the numbers of reactant species at the beginning of the reaction. Current algorithms follow the time evolution of a well-stirred chemically reacting system subject to molecular noise. These algorithms are able to successfully tackle the complexities of several dynamic systems in oncology, including gene regulatory networks and tumor suppressor pathways [11–14].

The breast cancer stem cell niche represents a complex system where multiple pathways regulate the behavior of the breast cancer stem cell, including whether it undergoes self-renewal, quiescence, differentiation, or apoptosis. Discoveries in recent years suggest that differentiated epithelial cells in normal breast and breast cancer tissues may have the ability to dedifferentiate into stem-like cells, and this plasticity has important implications for targeting cancer stem cells [15–17]. Existing stochastic models that quantify rates of conversion of differentiated cells to a stem-like state [18] do not take into consideration the effects of the stem cell environment or the existence of different cancer stem cell states. We have recently reported that BCSCs display phenotypic plasticity enabling them to interconvert between a rapidly proliferative state (epithelial or MET-like state) marked by ALDH expression and an invasive quiescent state (mesenchymal or EMT-like state) marked by CD44+/CD24- expression [19]. Furthermore, CSC state transitions are regulated by components in the tumor microenvironment which in turn regulate intracellular signaling pathways and microRNAs in the CSC population [19–22]. Stochastic simulation is ideal for examining the rates and regulators of EMT/MET transitions and predicting response to therapy.

Stochastic models have proven useful in the study of population dynamics in cancer stem cells, where the cancer stem cell population is small relative to the total tumor cell population, and the events of interest, such as extinction and mutation, are rare events [5, 7]. Using stochastic models, we can estimate full distributions of cancer stem cell counts over time throughout the duration of different therapeutic combinations, and quantify the variance in these cell counts when they drop to critically small numbers. Quantifying the frequency of the event that the cancer stem cell population goes extinct using stochastic simulation allows us to examine the conditions under which we would expect the cancer stem cell population to be eradicated with therapy.

We construct a model that incorporates intracellular signals and microenvironmental factors to describe population dynamics of breast cancer stem cells during carcinogenesis and in response to treatment. Compelling questions we address with modeling include: (1) What is the rate of transition between the epithelial and mesenchymal states? (2) How does a rarely dividing cancer stem cell eventually lead to rapid tumor growth? (3) What are the key components of niche regulation, and what are the effects of blocking each component individually and in combination? We examine whether our predictions from our modeling match experimental data on breast tumor growth. Finally, we describe combinations of therapy that optimally exploit breast cancer stem cell properties. We find that blocking the inflammatory positive feedback loop between IL-6 and NF-*κ*B involved in *β*-catenin signaling and BCSC self-renewal is required to effectively target both mesenchyaml and epithelial BCSCs, and our predictions match experimental findings showing the combined efficacy of IL-6 and HER2 blockade [19].

## Materials and Methods

### Estimation of transition rates

Breast cancer stem cells exist in two freely interconvertible states: the mesenchymal state, and the epithelial state. In this section, we adopt a simple model for the purposes of estimating rates of transition between these two states. To estimate the transition probabilities for the EMT and MET, we model the breast cancer stem cell population as a continuous-time Markov chain with two states. A two-state continuous-time Markov chain always satisfies the detailed balance condition *π*_1_ *λ*_12_ = *π*_2_ *λ*_21_, where *π* = (*π*_1_, *π*_2_) is the equilibirum distribution for the chain, and *λ*_12_ and *λ*_21_ are transition rates between the two states [23]. Based on experimental observations that in the triple negative breast cancer SUM159 cell line the ALDH+ cell population as assessed by the Aldefluor assay constitutes roughly 4% of breast cancer cell populations, 1% of cells are tumor-initiating, and nearly all cells are CD44+CD24- [20], we estimate the equilibrium distribution of the mesenchymal state (*π*_*EMT*_) to be 0.2, and that of the epithelial state (*π*_*MET*_) to be 0.8. In contrast, in MCF-7 cell line experiments, where cells are derived from a more indolent luminal breast cancer, 0.3% of cells are ALDH+ and 0.8% of cells are CD44+CD24-, and $\pi_{EMT}=\frac{0.008}{0.008+0.003}=0.73$. The theoretical formula:

$$\begin{array}{c} \pi_{MET}=\frac{\lambda_{MET}}{\lambda_{EMT}+\lambda_{MET}}, \end{array}$$

expresses *π*_*MET*_ in terms of *λ*_*MET*_, the rate of conversion from a mesenchymal to an epithelial state, and *λ*_*EMT*_, the rate of conversion from an epithelial to a mesenchymal state. In the first scenario we accordingly estimate *λ*_*MET*_ to be 4 × *λ*_*EMT*_.

### Regulators of transition

**Fig 1:**
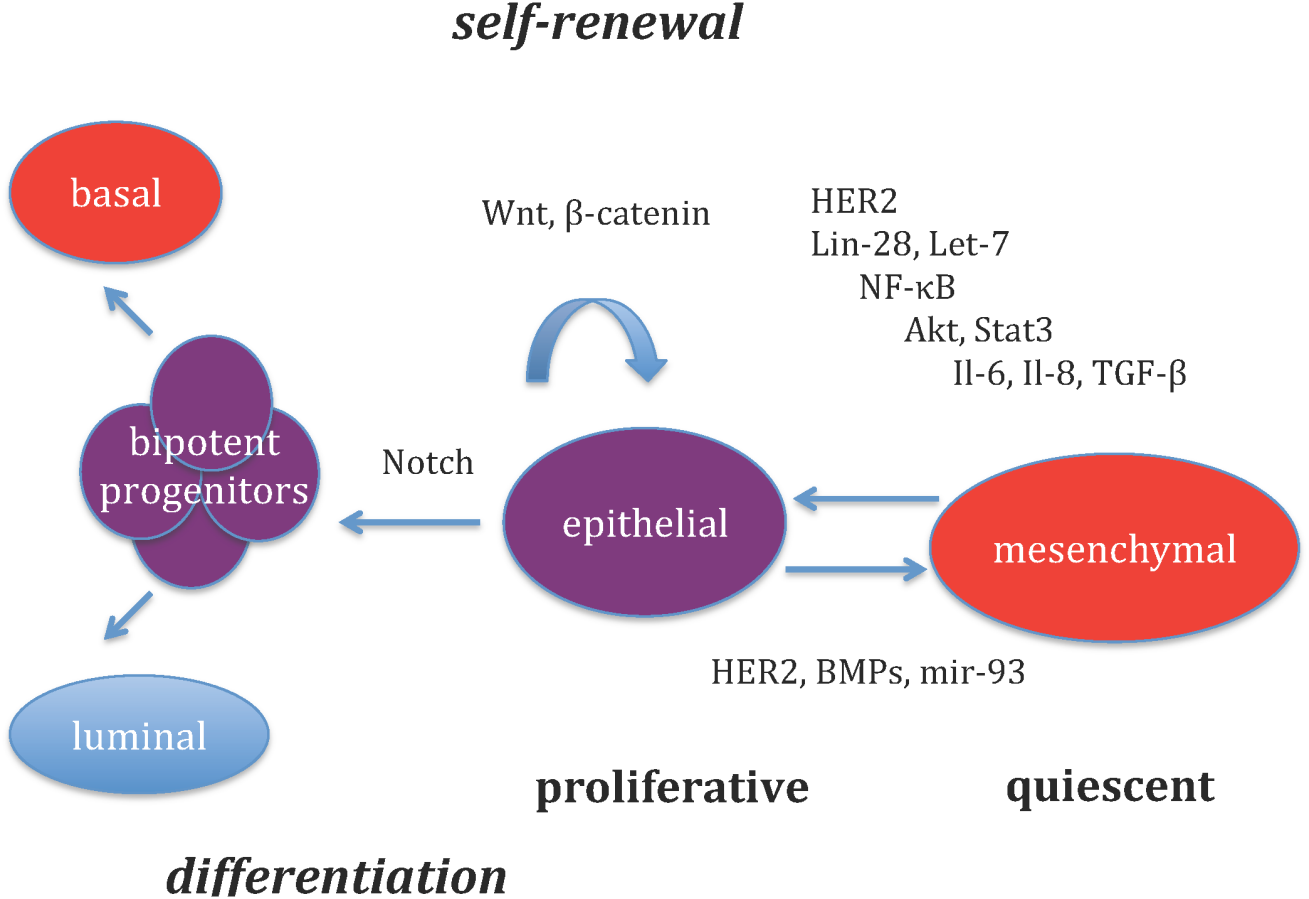
Players in the breast cancer stem cell niche. Breast cancer stem cells (BCSCs) readily interconvert between two states: a highly proliferative MET-like state marked by ALDH+ expression and expression of epithelial markers, and an EMT-like state which is CD44+, quiescent, expresses mesenchymal markers, and is capable of tissue invasion and metastasis. Conversion to a mesenchymal state is promoted by IL-6 and TGF-*β*, while conversion back to an epithelial state is enhanced by BMPs, HER2, and mir-93 expression. BCSC self-renewal is enhanced by *β*-catenin signaling, which is activated by HER2 and Lin-28. Both Lin-28 and IL-6 are inhibited by Let-7. Akt is activated by HER2 and IL-8, while Stat3 is activated by IL-6 and IL-8 receptor binding. Activation of Akt and Stat3 both lead to activation of NF-*κ*B signaling, increasing expression of Lin-28 and IL-6, leading to a positive feedback loop.

The BCSC niche governs whether BCSC remain in a quiescent state (mesenchymal) or enter into a proliferative state (epithelial), where they can undergo self-renewal and differentiation. Fig 1 depicts key regulators that modulate the interconversion between these two states. Cytokines, including IL-6 and TGF-*β*, have been shown to drive EMT [19]. Inflammation activates NF-*κ*B, which in turn drives IL-6 production, generating a positive feedback loop [19]. Loss of PTEN is associated with HER2-targeted therapy resistance, and this association is mediated by Akt with subsequent activation of NF-*κ*B sustaining an IL-6 inflammatory loop [19]. MicroRNAs, including mir-93, regulate the mesenchymal to epithelial transition as well as proliferation and differentiation of BCSCs [20].

We can examine values of *λ*_*EMT*_ and *λ*_*MET*_ in response to regulators, including intracellular signals such as miRNAs and microenvironmental cytokines such as IL-6. We use data on changing patterns of mRNA expression of vimentin and E-cadeherin in pTRIPZ-SUM159-mir-93 cells plated with doxycycline to induce mir-93 expression [20]. Because induced mir-93 expression drives MET [20], we fit a declining exponential curve to the level of vimentin expression to estimate an upper bound to the rate of MET conversion. Based on these calculations, we estimate the rate of transition from a mesenchmal to epithelial state to be 0.08 cell^−1^ day^−1^, and therefore the rate of transition from an epithelial to a mesenchymal state to be 0.02 cell^−1^ day^−1^.

Loss of PTEN is associated with activation of an IL-6 inflammatory feedback loop with 10- to 20-fold increases in cytokine levels [19]. Fitting an exponential curve to the increasing level of vimentin expression in BT474PTEN^−^ cells, we estimate the rate of conversion from an epithelial to a mesenchymal state to be 0.46 cell^−1^ day^−1^ when IL-6 levels are very high.

Our estimated rates of EMT/MET interconversions are much faster than the rate of dedifferentiation estimated from human iPS cell experiments [24]. In that setting, fibroblasts are reprogrammed and cultured for 28 days, and approximately 1% of cells are successfully transformed ≈ 0.0036 cell^−1^ day^−1^. These results suggest that stem cell state transitions are far more frequent than the event of dedifferentiation.

### Step anticipation *τ*-leaping

Stochastic simulation was introduced in 1976 by Daniel Gillespie to generate exact trajectories of a stochastic equation in order to accurately simulate chemical or biochemical systems with small numbers of molecules [25]. In the stochastic simulation framework, the process is formulated as a continuous-time Markov chain, where the state vector *X*(*t*) = (*X*1(*t*), …, *XN*(*t*)) providing the number of molecules of each species at time t, evolves stochastically. Random collisions driven by the inherent randomness of thermal molecular motion give rise to random state transitions specified by a set of reaction channels, and the waiting times before transitions between states follow an exponential distribution [25]. In the algorithm, reactant species and a set of events or ‘reactions’ are specified. For each reaction *j*, a propensity *r*_*j*_(*X*_*t*_) is defined, which is dependent on the current counts of each species involved in the reaction, as well as the reaction rate constant *a*_*j*_. A stoichiometric matrix denotes the discrete amount each reaction species changes with each reaction. This algorithm has been successfully applied to many problems in computational systems biology. However, because the algorithm involves simulation of every reaction that occurs, it can be slow for large and increasingly complex systems.

Tau-leaping algorithms allow for increased speed by leaping over a set of reactions within a small time interval, but can reduce accuracy if the reaction propensities change considerably during the leap [26]. In tau-leaping, we move through the continuous-time Markov chain by leaping over a time period (*t*, *t*+*τ*), where we make the assumption that the number of reactions of type *j* that occur during the interval (*t*, *t*+*τ*) is Poisson distributed with mean set to the reaction propensity [26]. Provided that the size of the leap is small enough so that the propensity function does not change more than a certain amount that depends on a pre-specified error control parameter, the probability for an event to occur in the next infinitesimal time *dt* is *a* ⋅ *dt* where *a* is constant, and we can estimate the state change during the leap by Poisson sampling [26]. We run an independent Poisson process for each reaction channel and sum the results.

We employ the step anticipation *τ*-leaping (SAL) algorithm in our simulations because of its increased accuracy without compromising speed [27]. In SAL, we expand each intensity using a Taylor’s series around time *t* with base value *r*_*j*_(*X*_*t*_). Let *c* denote the number of reaction channels and *d* denote the number of reactant species. The number of reactions of type *j* is sampled from a Poisson distribution with mean

$$\begin{array}{ccc} \omega_{j}\left(t,t+\tau \right) & = & \int_{0}^{t}[r_{j}\left(X_{t}\right)+\frac{d}{dt}r_{j}\left(X_{t}\right)s]ds\\ & = & r_{j}\left(X_{t}\right)\tau +\frac{d}{dt}r_{j}\left(X_{t}\right)\frac{1}{2}\tau^{2}. \end{array}$$

We start with the reaction rate equation (RRE) that models the mean behavior *μ*(*t*) = 𝔼(*X*_*t*_) of the system using a system of ordinary differential equations:

$$\begin{array}{c} \frac{d}{dt}\mu \left(t\right)=\sum_{j=1}^{c}r_{j}\left[\mu \left(t\right)\right]\nu^{j}, \end{array}$$

where *ν* ^*j*^ is the increment vector for reaction *j*. In order to obtain the time derivative of *r*_*j*_(*X*_*t*_), we use the chain rule:

$$\begin{array}{c} \frac{d}{dt}r_{j}\left(x\right)=\sum_{k=1}^{d}\frac{\partial }{\partial x_{k}}r_{j}\left(x\right)\frac{d}{dt}x_{k}, \end{array}$$

and set

$$\begin{array}{c} \frac{d}{dt}x_{k}=\sum_{j=1}^{c}r_{j}\left(x\right)\nu_{k}^{j} \end{array}$$

using the mean behavior of the system as calculated using the RRE. SAL increases accuracy of simulations without compromising speed, allowing the simulation to proceed with larger steps [27]. The improved accuracy is more pronounced in systems in more complex models with higher order kinetics that entail more rapidly changing intensities.

### Stochastic simulation of the BCSC niche

While the intial model described above includes only two stem cell states, more sophisticated models are required to study the effects of the microenvironment on breast cancer stem cell behavior. We employ stochastic reaction kinetics techniques to study population dynamics of breast cancer stem cells and their progeny. Our model is schematically depicted in Fig 1. Breast cancer stem cells (BCSCs) readily interconvert between a quiescent, invasive, mesenchymal state, and a proliferative epithelial state. While in the proliferative epithelial state, a BCSC can undergo symmetric self-renewal giving rise to two identical epithelial BCSCs, or asymmetric self-renewal, giving rise to one epithelial BCSC, and one bipotent progenitor (BPP). BCSCs in the epithelial state can also undergo symmetric differentiation, giving rise to two BPPs. In addition the epithelial BCSCs are susceptible to apoptosis. Bipotent progenitors either symmetrically divide, giving off two progenitors, or differentiate into luminal or basal cells. Cytokines, including Il-6 and TGF-*β* promote the conversion from an epithelial state to a mesenchymal state [19]. MicroRNAs, such as mir-93 promote the awakening of quiescent BCSCs from a mesenchymal to an epithelial state, in part by inhibiting TGF-*β* signaling [20]. BMPs and HER2 signaling also enhance the transition from a mesenchymal to an epithelial state [28, 29]. In addition, Il-6 promotes Stat3 activation, and Il-8 promotes Akt and Stat3 activation, both of which activate NF-*κ*B. Activated NF*κ*B leads to expression of Lin28, which activates *β*-catenin signaling, which drives self-renewal of epithelial BCSCs. In addition, activated NF*κ*B increases transcription of Il-6, leading to a positive feedback loop with increased Stat3 and NF*κ*B activation. Lin28, an important stem regulatory gene, decreases cellular differentiation and increases self-renewal, through downregulation of Let-7, a microRNA that limits capacity for self-renewal [30]. Lin28 is associated with overexpression of HER2 and sustains the inflammatory feedback involving IL-6 and NF-*κ*B. In addition to promoting self-renewal, Lin28 increases transcription of the Her2 receptor. Dimerization of the Her2 and EGFR leads to Akt activation. Let-7 inhibits Lin28, thereby inhibiting self-renewal, and in addition inhibits Il-6. In the stochastic reaction kinetics framework, we define our model by listing reactant species, reactions (or events), and reaction propensities.

### Parameters and initial conditions

Parameter values are drawn from the literature when possible and otherwise from reasonable estimates based on the experimental evidence available. Rates of symmetric self-renewal are taken from extrapolations from feline and murine experiments on hematopoietic stem cells [31]. Asymmetric self-renewal is presumed to occur much more frequently than symmetric self-renewal, while symmetric differentiation occurs at the same infrequent rate as symmetric self-renewal [32, 33]. Receptor binding and dissociation occur on similar time scales, while transcriptional events are more rare [8]. S1 Table lists the rate constant for each reaction. We explore sensitivity of our predictions to varying the death rate of epithelial BCSCs and the rate of epithelial to mesenchymal transition (EMT).

At the beginning of each simulation, we set the particle count equal to 800 for epithelial BCSCs, and 200 for mesenchymal BCSCs. These estimates are based on our calculations for relative frequencies of each particle type taken from xenograft experiments. Initial particle counts for cytokines IL-8 and TGF-*β* were set equal to 100, while initial counts for IL-6 were set to 1000 to explore the scenario where the ratio of IL-6 to other cytokines is elevated in breast tumors. While in reality we would expect the number of molecules of cytokines (≈ 10^8^/ml) to far outweigh the tumor cell count (≈ 10^3^/ml), we made the simplifying assumption that all 37 reactions were occurring on similar time scales and with propensities of similar magnitudes. We made this assumption in order to explore the impact of inhibiting cytokine and intracellular signals, both individually and in combination, by decreasing the rate constant of each reaciton. Initial counts for receptors, including gp130, HER2, EGFR, CXCR1, and TGF*β*R2, and signaling molecules, including Akt, I*κ*B⋅p50⋅RelA, Let-7, *β*-catenin, mir-93, BMP, Stat3, HER2 mRNA, were set to 100. Counts for all signal-receptor dimers (e.g. Il-6 ⋅ gp130) and activated signaling molecules (e.g. activated Stat3), as well as Lin-28, were initially set to 0.

In order to simulate a situation where a reaction is inhibited, we maintain the same initial conditions, and change the reaction rate constant. The new reaction rate is obtained by multiplying the rate constant of the reaction by 10^−10^.

## Results

### Stem cell niche regulation and Gompertzian growth kinetics

The cancer stem cell hypothesis asserts that tumor growth and invasion are driven by the cancer stem cell population. This suggests that bulk tumor growth kinetics depend heavily on relatively small changes in the behavior of cancer stem cells. We explore the effects of varying the rate of symmetric self-renewal in BCSCs, while holding the rates of division, differentiation, and death constant in the progenitor and differentiated cell populations.

Self-renewal can be symmetric, giving rise to two BCSCs, or asymmetric, giving rise to one BCSC and one partially differentiated daughter cell. The fate of a BCSC is highly regulated by microenvironmental signaling, including cytokines as well as intracellular signaling and miRNAs. The predominant mode of healthy stem cell self-renewal is asymmetric, but the proportion of stem cells undergoing symmetric self-renewal may increase over time during carcinogenesis. A recent study showed that asymmetric segregation of template DNA varies by molecular breast cancer subtype and several factors can alter the frequency of asymmetric segregation in breast cancer [33]. Studies tracking multiple cell divisions from initial single cells show that symmetric division is the predominant (84.6%) mode of division in Oct4+ breast cancer cells [34]. Further studies of mammary stem cells in mammospheres demonstrate that p53 regulates polarity of cell division in mammary stem cells and suggest that loss of p53 favors a shoft towards symmetric division of cancer stem cells [35]. We accordingly consider a model in which the rate of symmetric self-renewal in breast cancer stem cells gradually increases during carcinogenesis. Breast tumors have been shown to follow a Gompertzian curve [36–38], a sigmoid function where growth is slowest at the start and end of a time period, and the future value asymptote of the function is approached much more gradually than the lower valued asymptote. We demonstrate here how Gompertzian growth kinetics can be explained by small alterations in rates of symmetric versus asymmetric self-renewal.

We explore tumor growth dynamics under a gradually increasing percentage of symmetrically dividing stem cells. We make the assumption that the initial rate of symmetric self-renewal is slow and upon transformation to a breast cancer stem cell the rate of symmetric self-renewal steadily increases [34, 35, 39]. Asymmetric self-renewal is the predominant mode of self-renewal in healthy stem cells, with observations of 80% of WT mammmary cells dividing asymmetrically [35], and we make the assumption that the remainder of time is split evenly between symmetric self-renewal and symmetric differentiation. Extrapolations from feline and murine data for hematopoietic stem cells indicate that symmetric self-renewal occurs approximately once every 42 weeks (*β* = 0.0034 cell^−1^ day^−1^). We hypothesize that the rate of asymmetric self-renewal is accordingly much faster (*α* = 0.027 cell^−1^ day^−1^). Symmetric differentiation is occurring at the same slow rate of symmetric self-renewal (*ρ* = 0.0034 cell^−1^ day^−1^). Studies tracking multiple cell divisions from initial single cells show that symmetric division is the predominant (84.6%) mode of division in Oct4+ breast cancer cells [34]. Further studies of mammary stem cells in mammospheres demonstrate the p53 regulates polarity of cell division in mammary stem cells and suggests that loss of p53 favors a shift towards symmetric division of cancer stem cells [35]. Loss of p53 is associated with an increase in the frequency of symmetric divisions among mammospheres, while pharmacological restoration of p53 leads to restoration of asymmetric division as the predominant mode of division [35]. Upon malignant transformation, there is an observed increase in symmetric stem cell divisions to 78% [35]. Based on these findings, we examine tumor growth dynamics in response to a gradual increase in breast cancer stem cell symmetric division from 20% to 80% over several years.

We begin with a population of 25 BCSCs, 5 in the quiescent mesenchymal state and 20 in the rapidly proliferating epithelial state, as well as 100 bipotent progenitors, and 500 differentiated cells. In each scenario, we gradually increase the frequency of symmetric divisions in BCSCs from 20% to 80% over a period of 6 years and follow population dynamics out to 9 years. We hold the rate of asymmetric self-renewal constant (0.027 cell^−1^ day^−1^) and increase the rates of symmetric self-renewal (*β*) and symmetric differentiation (*ρ*). The frequency of symmetric division is increased yearly (20% in year 1, 30% in year 2, 40% in year 3, 50% in year 4, 60% in year 5, 70% in year 6, 80% in years 7 through 12). The frequency of asymmetric division accordingly decreased yearly (80% in year 1, …, 20% in years 7 through 9). We allow the frequency of symmetric self-renewal to slightly exceed that of symmetric differentiation and follow the predicted total tumor cell population.

**Fig 2:**
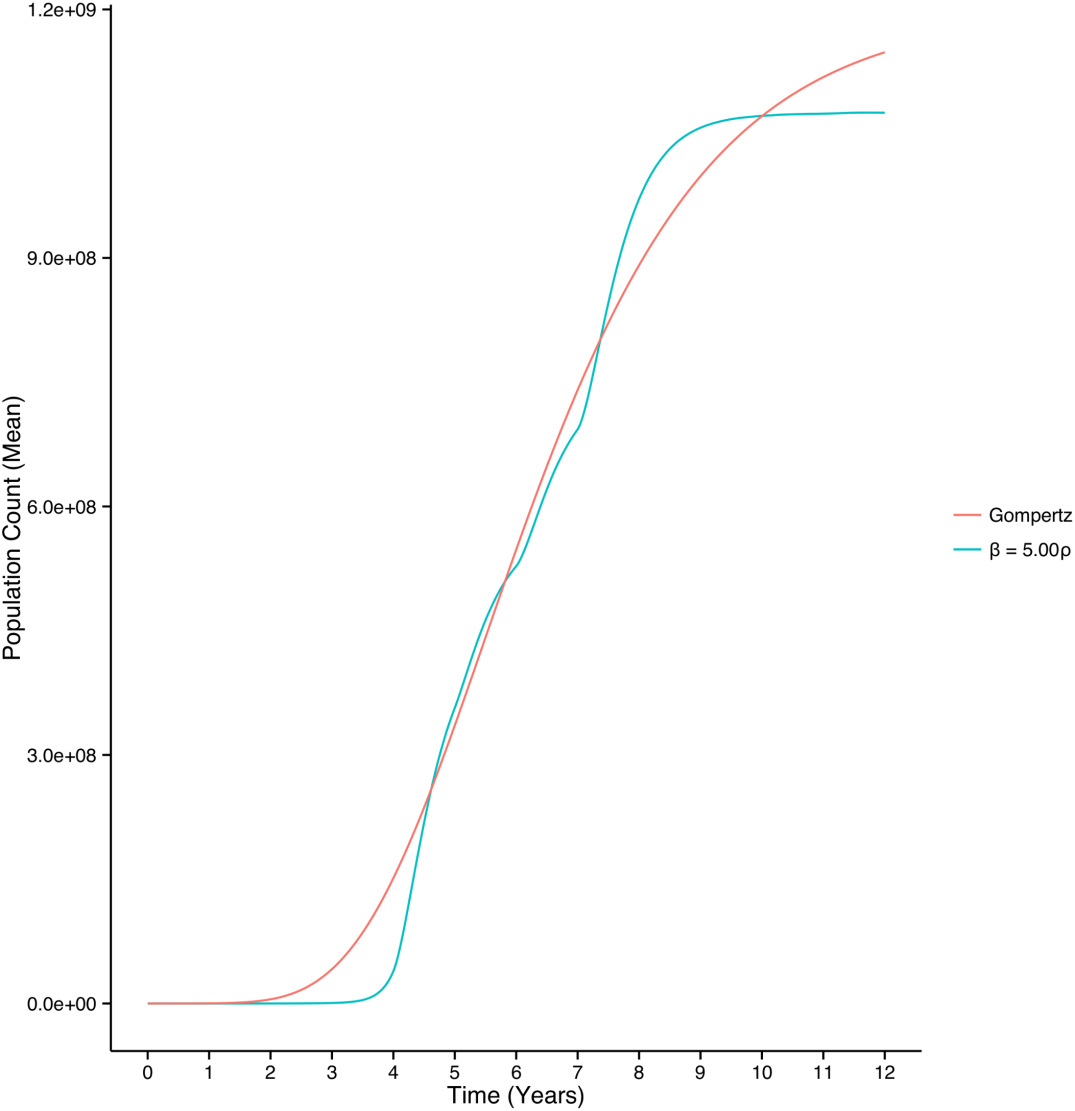
Stochastic simulation results for cell growth during carcinogenesis compared with analytic results for the Gompertzian growth curve. Simulation results show total population counts in response to a gradual shift from predominantly asymmetric to symmetric division of BCSCs. Our simulation showed excellent agreement with the Gompertz function with growth rate parameter *c* ranging between 0.001 and 0.002 cell^−1^ day−1, which match previously estimated growth rate parameters obtained by fitting the Gompertz function to data from breast tumor growth [37, 38]. Here we demonstrate the fit between our simulation results and the Gompertz function with parameters *A*_0_ = 0.0193 (95% confidence interval 0.0192—0.0194), *c* = 0.00133 (95% confidence interval 0.00133-0.00134) and asymptote 1.2 × 10^9^ (*R* ^2^ = 0.989).

Fig 2 shows the mean trajectory of the total cell population in response to a shift from asymmetric to symmetric division. We used coarse variations for the ratio between *β* and *ρ* in different time periods and used least squares to estimate the fit between our predicted total tumor cell population trajectories and tumor growth data from breast tumors [37, 38]. We found that letting *β* = 5 × *ρ* during years 1–4 and *β* = *ρ* during years 5–12 provided the best fit. We note that the predicted total tumor cell population resembles closely the shape of Gompertzian growth kinetics, where there is continuous deceleration of tumor growth [36]. We were able to estimate fit between our simulation results and the Gompertz function *N*(*t*) = exp[(*A*_0_/*c*)(1−exp(−*ct*)] while varying the initial specific growth rate *A*_0_ from 0.02 to 0.04, and the proportional rate of decay *c* from 0.001 to 0.002. Parameter values previously estimated for the proportional rate of decay using data from human breast tumors fall within this range [37]. For the simulation shown in Fig 2, we find that the total tumor growth matches observed Gompertzian tumor growth kinetics, where a tumor takes 8 years to grow from a single cell to clinical recognition at 10^9^ cells [37, 38]. We obtained excellent fit (*R* ^2^ = 0.99) between the Gompertz curve and our simulation when *c* = 0.0013 and *A*_0_ = 0.027.

**Fig 3:**
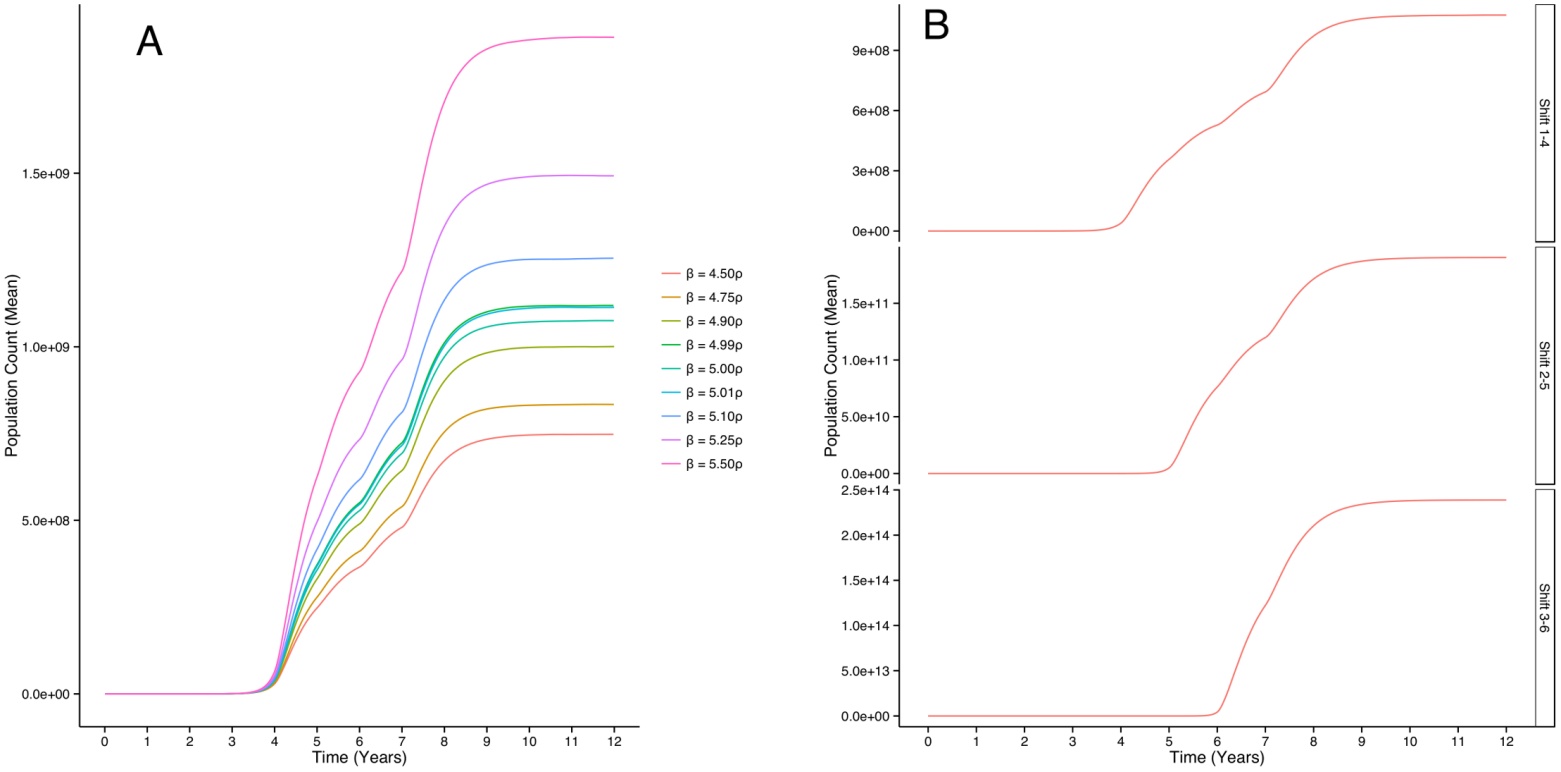
Average trajectories of total cancer cell populations in response to varying the difference between symmetric self-renewal (*β*) and symmetric differentation (*ρ*) rates and the time period during which *β* diverges from *ρ*. Higher peak difference ∣*β*−*ρ*∣ leads to a higher asymptotic population size (panel A). Allowing *β* to diverge from *ρ* later in the course of carcinogenesis translates the Gompertz curve to the right and delays the growth of the tumor, while increasing its ultimate size (panel B).

Next, we explore the effects of increasing the difference between symmetric self-renewal rates and symmetric differentiation rates. In each case we allow the frequency of symmetric self-renewal to exceed that of symmetric differentiation in years 1 through 4. Panel A of Fig 3 shows the effect of varying this peak difference on the total cancer cell population size. As we increase the difference between the frequencies of symmetric self-renewal and symmetric differentiation (*β* = 4.5*ρ* to *β* = 5.5*ρ*), we note that the asymptotic population sizes are increased.

Finally, we show that varying the time period during which symmetric self-renewal exceeds symmetric differentiation affects both the rate of continuous deceleration of tumor growth and the asymptotic population size. Panel B of Fig 3 reveals that delaying the point at which *β* exceeds *ρ* shifts the curve to the right, delaying bulk tumor growth, but increases the asymptotic population size.

We find that the continuous deceleration that is characteristic of Gompertzian growth is observed whenever both of the following two conditions are met: 1) there is a shift from asymmetric to symmetric division in BCSCs, and 2) the rate of symmetric self-renewal initially exceeds and then approaches the rate of symmetric differentiation. S1 Fig reveals the effects of relaxing either of these requirements. In both cases, there is not appreciable expansion of the stem cell compartment, and while slow tumor growth occurs, the continuous deceleration of Gompertzian growth is not observed.

We conclude that small perturbations in breast cancer stem cell behavior can lead to Gompertzian growth kinetics. In our model, progenitor division rates are held constant, and we do not make assumptions about limited resources for cancer cell growth.

### Targeting the BCSC microenvironment

The behavior of cancer stem cells is highly regulated by signaling in the microenvironment, and targeting the microenvironment may be an effective way of eradicating cancer stem cells. We explore the population dynamics of BCSCs under therapy targeting microenvironmental signaling. Specifically, we identify changes in cell population dynamics in response to inhibition of niche regulators, knocking them out individually and in combination.

Stochastic reaction kinetics is ideal for studying the complex regulatory dynamics of the BCSC niche. Table 1 lists reactant species involved in our model system. These species include the EMT-like and MET-like BCSC populations, as well as the levels of cytokines (IL-6, NF-*κ*B, TGF-*β*), and molecular regulators (HER2, mir93) of self-renewal and EMT/MET transitions. Table 2 lists the events that occur in our system. An example of a positive feedback loop involving IL-6 and NF-*κ*B is shown in bold.

**Table 1:** Reactant species in the BCSC niche model. Examples of reactants include cell populations, microenvironmental regulators such as cytokines, and intracellular signals such as microRNAs.

*Cells*	*Cytokines*	*Receptors*
MET-like BCSC	IL-6	gp130
EMT-like BCSC	IL-8	CXCR1
	TGF-*β*	HER2
	BMP	EGFR
**Complexes**		
IL-6 ⋅ gp130 dimer	IL-8 ⋅ CXCR1	HER2 ⋅ EGFR
Lin-28 ⋅ HER2 mRNA	I*κ*B ⋅ p50 ⋅ RelA	Let-7 ⋅ IL-6
Lin-28 ⋅ Let-7	mir93 ⋅ TGF-*β*	BMP
***Intracellular signals***		
Stat3	activated Stat3	HER2 mRNA
Akt	activated Akt	I*κ*B
p50 ⋅ RelA	Lin-28	Let-7
*β*-catenin	activated *β*-catenin	mir-93

**Table 2:** Reaction channels of the BCSC niche system. Events include self-renewal and death of BCSCs, dimerization of ligand and receptor and their dissociation, activation and inhibition of signaling molecules, and increased transcription in the presence of activating signals. Bolded reactions represent those involved in the inflammatory feedback loop between IL-6 and NF-*κ*B. IL-6 binds to its receptor gp130, leading to Stat3 activation, which causes NF-*κ*B activation. Activated NF-*κ*B increases expression of IL-6, leading to a positive feedback loop, as well as increased expression of Lin-28, which activates *β*-catenin signaling. Let-7 directly inhibits both Lin-28 and IL-6.

*Receptor binding and dissociation*	
**IL-6 + gp130 → IL-6 ⋅ gp130**	IL-8 + CXCR1 → IL-8 ⋅ CXCR1
IL-6 ⋅ gp130 → IL-6 + gp130	IL-8 ⋅ CXCR1 → IL-8 + CXCR1
TGF-*β* + TGF-*β*R2 → TGF-*β* ⋅ TGF-*β*R2	TGF-*β* ⋅ TGF-*β*R2 → TGF-*β* +TGF-*β*R2
HER2 + EGFR → HER2 ⋅ EGFR	HER2 ⋅ EGFR → HER2 + EGFR
***Activation of intracellular signaling***	
**IL-6 ⋅ gp130 + Stat3 → IL-6 ⋅ gp130 + act Stat3**	IL-8 ⋅ CXCR1 + Akt → IL-8 ⋅ CXCR1 + act Akt
IL-8 ⋅ CXCR1 + Stat3 → IL-8 ⋅ CXCR1 + act Stat3	HER2 ⋅ EGFR + Akt → HER2 ⋅ EGFR + actAkt
actAkt + I *κ*B ⋅ p50 ⋅ RelA → act Akt + I*κ*B + p50 ⋅ RelA	actAkt → Akt
**act Stat3 + I *κ*B ⋅ p50 ⋅ RelA → act Stat3 + I*κ*B + p50 ⋅ RelA**	act Stat3 → Stat3
Lin-28 + HER2 mRNA → Lin-28 ⋅ HER2 mRNA	Lin-28 + *β*-catenin → Lin-28 + act *β*-catenin
HER2 ⋅ EGFR+ *β*-catenin → HER2 ⋅ EGFR+ act *β*-catenin	act *β*-catenin → *β*-catenin
Lin-28 + Let-7 → Lin-28 ⋅ Let-7	Lin-28 ⋅ Let-7 → Lin-28 + Let-7
Let-7 + Il-6 → Let-7 ⋅ IL-6	Let-7 ⋅ IL-6 → Let-7 + IL-6
mir93 + TGF-*β* → mir93 ⋅ TGF-*β*	mir93 ⋅ TGF-*β* → mir93 + TGF-*β*
I*κ*B + p50 ⋅ RelA → I*κ*B ⋅ p50 ⋅ RelA	
***EMT/MET transitions, self-renewal and cell death***	
MET + IL-6 ⋅ gp130 → EMT + IL-6 ⋅ gp130	MET + TGF-*β* → EMT + TGF-*β*
EMT + mir93 → MET + mir93	EMT + BMP → MET + BMP
EMT + HER2⋅ EGFR → MET + HER2 ⋅ EGFR	MET → 0
MET + act *β*-catenin → MET + MET + activated *β*-catenin	
***Transcription events***	
Lin-28 ⋅ HER2 mRNA → Lin-28 + HER2 + HER2 mRNA	**p50 ⋅ RelA → IL-6 + p50 ⋅ RelA**
p50 ⋅ RelA → p50 ⋅ RelA + Lin-28	

**Fig 4:**
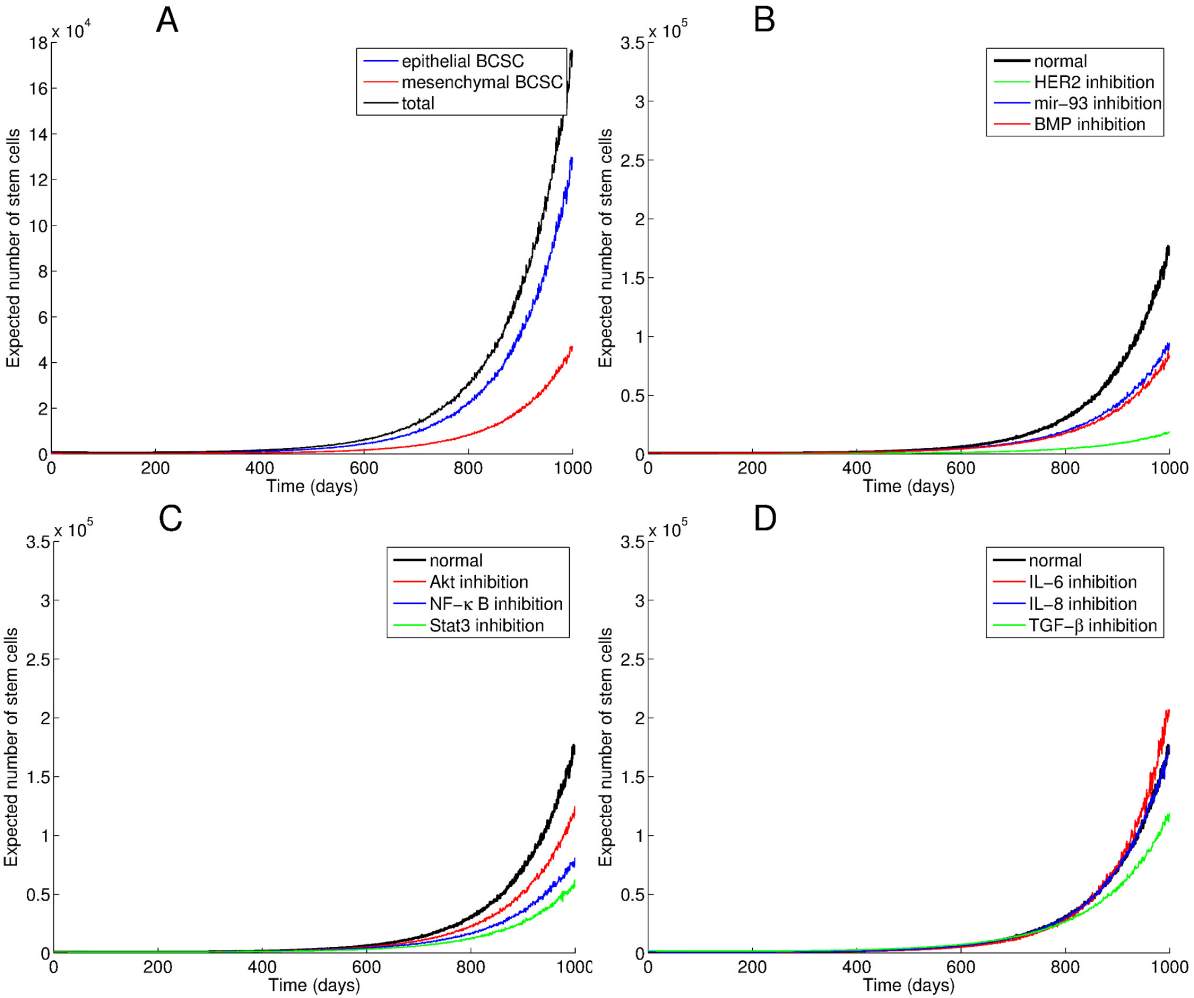
Predicted trajectories of BCSCs during carcinogenesis withoutout intervention (panel A) and in response to treatment (panels B-D). Population trajectories obtained by running 100 simulations for each scenario. Panel A reveals the trajectories of EMT-like and MET-like populations over time. In panels B-D, total BCSC counts are predicted after inhibiting elements of the BCSC niche one at a time. Inhibiting niche elements (IL-6 and TGF-*β*, panel D) that facilitate the transition from an MET-like to an EMT-like state causes an increase in the total population by increasing the proportion of proliferating cells, whereas inhibiting the reverse process (inhibiting HER2, mir-93, and BMP, panel B) causes an increase in the proportion of quiescent cells and a reduction in total BCSC counts.

Fig 4 reveals the trajectories of epithelial and mesenchymal BCSCs in the absence of therapy (Panel A) and under the effects of introducing inhibitors of several elements of the BCSC niche, including both intracellular signals and microenvironmental factors (Panels B-D). In particular these panels depict trajectories of predicted total BCSC counts in reponse to individually inhibiting elements of the BCSC niche. Each population trajectory reports average particle counts over 100 simulations. Panel A shows the effects of allowing the rate of EMT reactions (0.000225 cell^−1^ day^−1^) to exceed that of MET reactions (0.0001 cell^−1^ day^−1^). Shifting BCSC populations toward quiescence slows the growth of both populations. Targeting single elements of the BCSC niche reduces the rate of increase of BCSC populations, but the total BCSC population continues to rise in each case.

**Fig 5:**
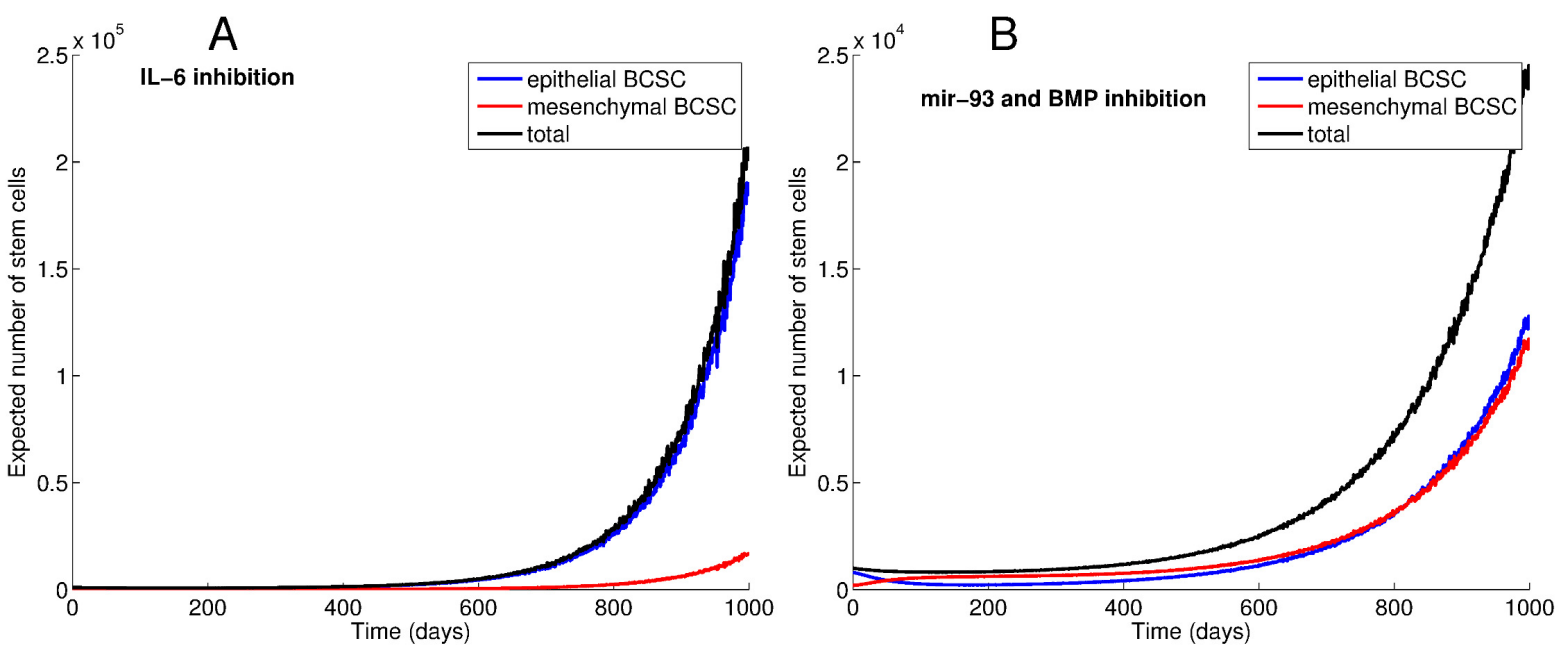
Trajectories of EMT-like and MET-like BCSC populations in response to niche targeted therapies. While blocking IL-6 leads to an increase in the MET-like BCSC population (panel A), causing more proliferation and a rapid increase in the total population size, simultaneously reducing mir-93 expression and inhibiting BMP causes a shift to the quiescent EMT-like state and a decrease in total BCSC counts (panel B).

Inhibiting IL-6 reduces the rate of conversion from an epithelial to a mesenchymal state, leading to an increase in the number of cells in the epithelial state (see the top panel of Fig 5). While inhibiting BMP and mir-93 expression leads to a decreased BCSC total count, it causes a shift to a higher proportion of invasive quiescent mesenchymal BCSCs (see the bottom panel of Fig 5), which may ultimately worsen prognosis.

**Fig 6:**
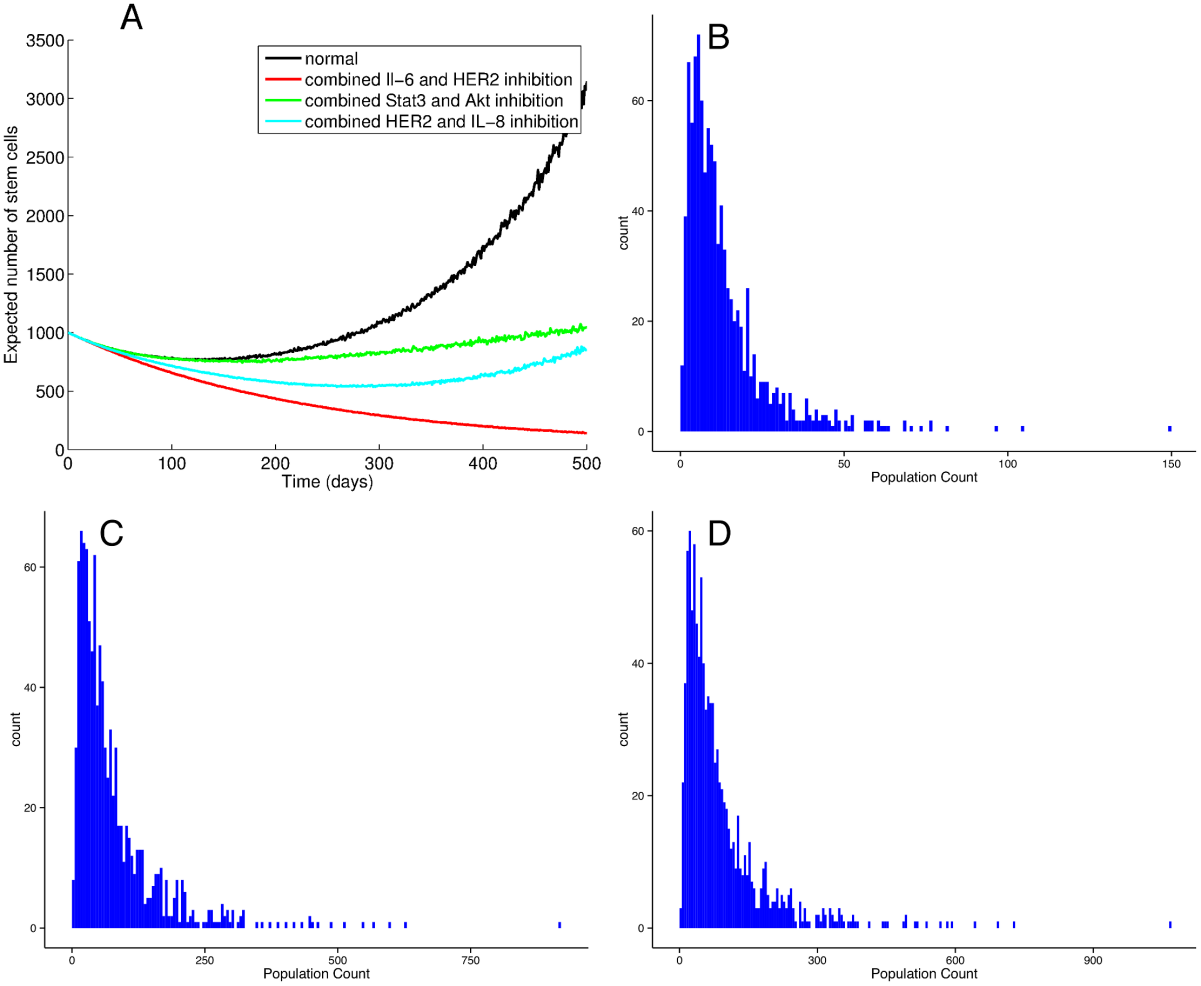
Combinations of therapies targeting the BCSC niche lead to delayed growth of the BCSC population. Population trajectories obtained by running 100 simulations for each scenario. The most effective combination of therapy is combined inhibition of HER2 and IL-6. By simultaneously reducing conversion to the quiescent, invasive EMT state, reducing proliferation of MET-like BCSCs, and inhibiting the inflammatory feedback loop, this combination leads to an effective elimination of both BCSC populations (panel A). Frequency distributions of species counts over 1000 simulations each run for 1000 days allow us to quantify how often cell counts reach zero under dual blockade of IL-6 and HER2 receptors, for the epithelial BCSC (panel B), mesenchymal BCSC (panel C), and total BCSC (panel D) populations.

When we examine the effects of combining inhibitors, we find that the most effective combination is simultaneous inhibition of HER2 and Il-6 (see Fig 6). Out of $\left(\begin{array}{c} 9\\ 2 \end{array}\right)=36$ combinations tried, this combination most effectively stops BCSCs from propagating. This occurs because of the effective blocking the inflammatory positive feedback loop between IL-6 and NF-*κ*B involved in *β*-catenin signaling and BCSC self-renewal. While other therapies, administered alone or in combination, are able to slow the growth of the BCSC population, this combination drives the BCSC population towards elimination.

Stochastic simulation allows us to further examine how frequently our BCSC populations are eradicated with therapy. Out of 1000 simulations, we plot the frequency distributions of epithelial BCSC, mesenchmal BCSC, and total BCSC population counts at the end of 1000 days of combined HER and IL-6 inhibitor therapy. We find that the BCSC are frequently eradicated by this optimal therapy.

**Fig 7:**
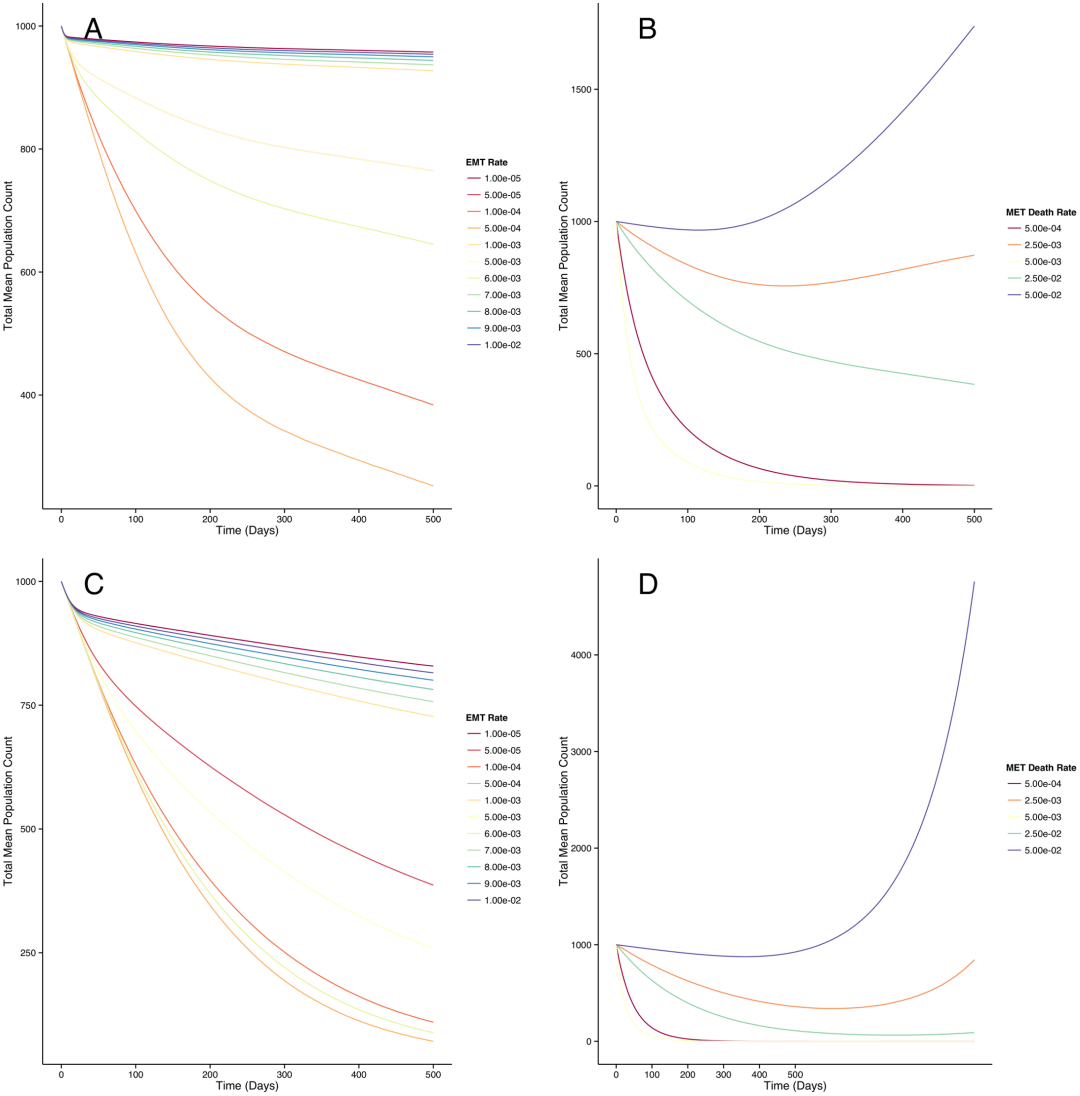
Effects of varying model parameters on predicted total BCSC population count trajectories. Under baseline conditions, increasing EMT slows growth of BCSC compartment (panel A) and smaller death rates of epithelial BCSC can lead to rapid growth of the BCSC compartment (panel B). When IL-6 and HER2 are simultaneously inhibited, the BCSC population is eradicated faster with slower rates of EMT (panel C) and higher epithelial BCSC death rates (panel D).

In order to examine the sensitivity of our results on the parameter values we chose, we examined the effects of varying our parameters both in the baseline niche and under the treatment combination we identified as most effective. Fig 7 shows the total BCSC counts over 1000 days in response to varying the rate of epithelial to mesenchymal transition (EMT) (panels A and C) and in response to varying the death rate of the proliferating epithelial BCSCs (panels B and D). Under baseline conditions (panels A and B), we find that decreasing the rate of EMT reduces the rate of growth of the total BCSC compartment, and smaller death rates of epithelial BCSCs leads to more rapid growth of the BCSC compartment. When combined IL-6 and HER2 inhibition are introduced (panels C and D), we find that the BCSC population is eradicated faster with slower rates of EMT and higher epithelial BCSC death rates.

## Discussion

We have developed a mathematical model to study key regulators of EMT and MET conversions in BCSCs and to predict response to therapies targeting these regulators. We examine the effects of therapies targeting the BCSC niche and conclude that the most effective combination of therapies to reduce the BCSC population involves the simultaneous inhibition of HER2 and the IL-6 inflammatory feedback loop. Our mathematical predictions show excellent agreement with experimental data from cell line and mouse xenograft studies showing the combined efficacy of HER2 and IL-6 blockade [19]. This is consistent with our previous studies supporting that the remarkable clinical efficacy of HER2 targets in the adjuvant setting may be due to the ability of these agents to effectively target CSCs [4]. These modeling results will be directly examined in a planned clinical trial of HER2 and IL-6 targeted therapy for breast cancer patients.

We find that the rate of interconversion between epithelial and mesenchymal BCSC states is much more frequent than estimates of the rate of de-differentiation. Earlier models have estimated more frequent rates of de-differentiation from in vitro and in vivo data [18]. However, these models did not take into account ALDH expression as a marker of stem cells, or the effects of the microenvironment. We estimate that the interconversion of stem cells between the EMT and MET-like states occurs frequently, and these transitions, rather than de-differentiation of non-stem cells, may account for the rapid conversion of CD44- populations to a CD44+ state. Our findings have important implications in assessing the degree of plasticity of breast cancer stem cells.

Our model highlights the dependence of the bulk tumor population on relatively small variations in BCSC dynamics. We explore the possibility that the Gompertz growth kinetics observed in most solid tumors can be explained by variations in the rate of symmetric self-renewal of BCSCs. While traditional interpretations of observed tumor growth patterns have attributed the deceleration to metabolic considerations, tumor-host interactions, and competition for resources as tumors grow larger [36], we propose that alterations in microenvironmental signaling of CSCs may account for the observed growth kinetics. This explanation is plausible, as recent evidence suggests that stem cell behavior is regulated by niche signaling, and that aberrant stem cell microenvironmental signaling contributes significantly to carcinogenesis [40]. By simply varying the percentage of BCSCs undergoing symmetric self-renewal, we predict bulk tumor growth patterns that match observed Gompertzian kinetics [38]. Microenvironmental signals such as HER2, *β*-catenin, and Lin-28, regulate symmetric self-renewal in breast cancer stem cells, and these signals are important during early stages of cancer growth. As tumors progress, symmetric self-renewal may diminish in order to maintain homeostasis in response to feedback from differentiated cells [41].

Interdisciplinary approaches combining theory and experiment and taking into account tumor heterogeneity and resistance to therapy have recently been implemented to optimize radiation dosing schedules [42]. Consideration of intratumoral heterogeneity and spatial effects of the BCSC niche, as well as incorporation of information on invasiveness of tumors would more accurately predict patient outcome. Future models should also include estimation of EMT/MET rates using patient tumor data rather than high passage cell lines. While therapies that promote the conversion to a quiescent state lead to diminished proliferation of BCSCs and slow growth of the BCSC population, the increased proportion of invasive cells would cause greater harm to the patient if metastatic foci deposits were more frequent. In addition, although mesenchymal BCSCs might contribute to tumor dormancy, these cells remain capable of transitioning to epithelial BCSCs accounting for recurrence at metastatic sites. In addition, our simulation models would be augmented by examining the effects of adding a differentiating agent such as retinoic acid (promoting asymmetric self-renewal and symmetric differentiation) to the system to determine the effect on extinction of BCSCs.

We have demonstrated here how a series of important biologically and clinically relevant questions in cancer stem cell research can be addressed successfully with mathematical modeling and simulations. Predictions from our simulations of niche target inhibitors match experimental data demonstrating the efficacy of combined HER2 and IL-6 blockade in pre-clinical models of HER2-positive breast cancer [19]. We anticipate that mathematical modeling in combination with experimental validation will optimize the development of safe and effective therapies that target the cancer stem cell and its niche. More broadly, our results suggest that elimination of CSC populations may require simultaneous targeting of epithelial and mesenchymal BCSC populations.

## Supporting Information

S1 Table Model parameters. S1 Table lists the parameters used in our model and simulation.
(PDF)

S1 Fig Exploring conditions required for Gompertzian growth. S1 Fig reveals the effects of allowing that no shift from asymmetric to symmetric division in BCSCs occurs (Panel A), and the rate *β* of symmetric self-renewal does not exceed the rate *ρ* of symmetric differentiation (Panel B) during carcinogenesis. In both scenarios, the continuous deceleration of Gompertzian growth is not oberved.
(TIFF)

S1 Appendix Population trajectories during carcinogenesis. S1 Appendix shows the predicted population trajectories for each cell species, including breast cancer stem cells (BCSCs), bipotent progenitors (BPP) and terminally differentiated cells (TCs) corresponding to the simulation results for total cell counts presented in Fig 2.
(PDF)

S2 Appendix S2 Appendix contains an implementation of our model in Systems Biology Markup Language (SBML) as requested by the reviewer. (PDF)

S3 Appendix S3 Appendix contains a table describing model fit. (XLSX)
