# Supplementary material for: Modeling of Cancer Stem Cell State Transitions Predicts Therapeutic Response
Source: PLoS One. 2015 Sep 23;10(9):e0135797. doi: 10.1371/journal.pone.0135797 (PMC4580445; doi:10.1371/journal.pone.0135797)
Supplement: S1 Table — lists the parameters used in our model and simulation. (PDF) [file pone.0135797.s001.pdf]

Supplementary Material

Table 3 lists the parameters used in our model and simulation.

**Reaction**	**Rate**
IL-6 + gp130 $\rightarrow$ IL-6 $\cdot$ gp130	10^{-4}
IL-8 + CXCR1 $\rightarrow$ IL-8 $\cdot$ CXCR1	10^{-5}
IL-6 $\cdot$ gp130 $\rightarrow$ IL-6 + gp130	8×10^{-3}
IL-8 $\cdot$ CXCR1 $\rightarrow$ IL-8 + CXCR1	8×10^{-3}
TGF-β + TGF-βR2 $\rightarrow$ TGF-β $\cdot$ TGF-βR2	10^{-4}
TGF-β $\cdot$ TGF-βR2 $\rightarrow$ TGF-β +TGF-βR2	8×10^{-3}
HER2 + EGFR $\rightarrow$ HER2 $\cdot$ EGFR	10^{-4}
HER2 $\cdot$ EGFR $\rightarrow$ HER2 + EGFR	10^{-2}
IL-6 $\cdot$ gp130 + Stat3 $\rightarrow$ IL-6 $\cdot$ gp130 + activated Stat3	10^{-4}
IL-8 $\cdot$ CXCR1 + Akt $\rightarrow$ IL-8 $\cdot$ CXCR1 + activated Akt	10^{-5}
IL-8 $\cdot$ CXCR1 + Stat3 $\rightarrow$ IL-8 $\cdot$ CXCR1 + activated Stat3	10^{-5}
HER2 $\cdot$ EGFR + Akt $\rightarrow$ HER2 $\cdot$ EGFR + activated Akt	10^{-4}
activated Akt + I κB $\cdot$ p50 $\cdot$ RelA $\rightarrow$ activated Akt + IκB + p50 $\cdot$ RelA	10^{-4}
activated Stat3 + I κB *cdot* p50 $\cdot$ RelA $\rightarrow$ activated Stat3 + IκB + p50 $\cdot$ RelA	10^{-4}
IκB + p50 $\cdot$ RelA $\rightarrow$ IκB $\cdot$ p50 $\cdot$ RelA	10^{-4}
Lin-28 + HER2 mRNA $\rightarrow$ Lin-28 $\cdot$ HER2 mRNA	10^{-4}
Lin-28 + β-catenin $\rightarrow$ Lin-28 + activated β-catenin	10^{-4}
HER2 $\cdot$ EGFR + β-catenin $\rightarrow$ HER2 $\cdot$ EGFR + activated β-catenin	10^{-4}
Lin-28 + Let-7 $\rightarrow$ Lin-28 $\cdot$ Let-7	10^{-4}
Lin-28 $\cdot$ Let-7 $\rightarrow$ Lin-28 + Let-7	8×10^{-3}
activated β-catenin $\rightarrow$ β-catenin	8×10^{-3}
p50 $\cdot$ RelA $\rightarrow$ p50 $\cdot$ RelA + Lin-28	8×10^{-3}
mir93 + TGF-β $\rightarrow$ mir93 $\cdot$ TGF-β	10^{-4}
mir93 $\cdot$ TGF-β $\rightarrow$ mir93 + TGF-β	8×10^{-3}
Let-7 $\cdot$ IL-6 $\rightarrow$ Let-7 + IL-6	8×10^{-3}
activated Akt $\rightarrow$ Akt	8×10^{-3}
activated Stat3 $\rightarrow$ Stat3	8×10^{-3}
Let-7 + Il-6 $\rightarrow$ Let-7 $\cdot$ IL-6	10^{-4}
MET + IL-6 $\cdot$ gp130 $\rightarrow$ EMT + IL-6 $\cdot$ gp130	10^{-4}
MET + TGF-β $\cdot$ TGF-βR2 $\rightarrow$ EMT + TGF-β $\cdot$ TGF-βR2	10^{-4}
EMT + mir93 $\rightarrow$ MET + mir93	10^{-4}
EMT + BMP $\rightarrow$ MET + BMP	10^{-4}
EMT + HER2 $\cdot$ EGFR $\rightarrow$ MET + HER2 $\cdot$ EGFR	10^{-4}
MET $\rightarrow$ 0	5×10^{-3}
MET + activated β-catenin $\rightarrow$ MET + MET + activated β-catenin	2×10^{-4}
Lin-28 $\cdot$ HER2 mRNA $\rightarrow$ Lin-28 + HER2 + HER2 mRNA	8×10^{-3}
p50 $\cdot$ RelA $\rightarrow$ IL-6 + p50 $\cdot$ RelA	8×10^{-3}

Table 3: Rates for each reaction used in our simulation. Rates are given in per species per day.
