# Supplementary figures and images for: Modeling of Cancer Stem Cell State Transitions Predicts Therapeutic Response
Source: PLoS One. 2015 Sep 23;10(9):e0135797. doi: 10.1371/journal.pone.0135797 (PMC4580445; doi:10.1371/journal.pone.0135797)

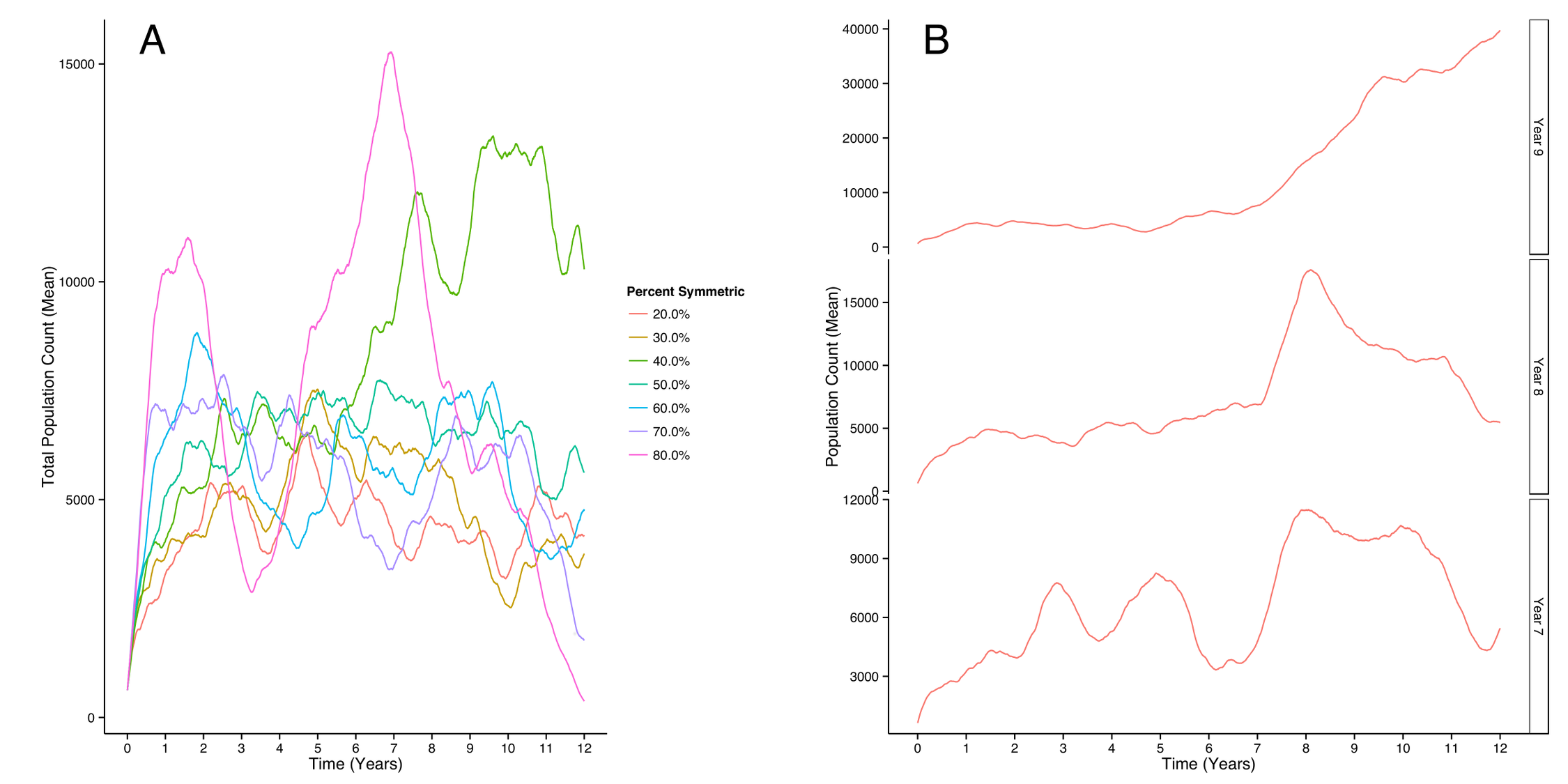

Supplement: S1 Fig — reveals the effects of allowing that no shift from asymmetric to symmetric division in BCSCs occurs (Panel A), and the rate β of symmetric self-renewal does not exceed the rate ρ of symmetric differentiation (Panel B) during carcinogenesis. In both scenarios, the continuous deceleration of Gompertzian growth is not oberved. (TIFF) [file pone.0135797.s002.tiff]

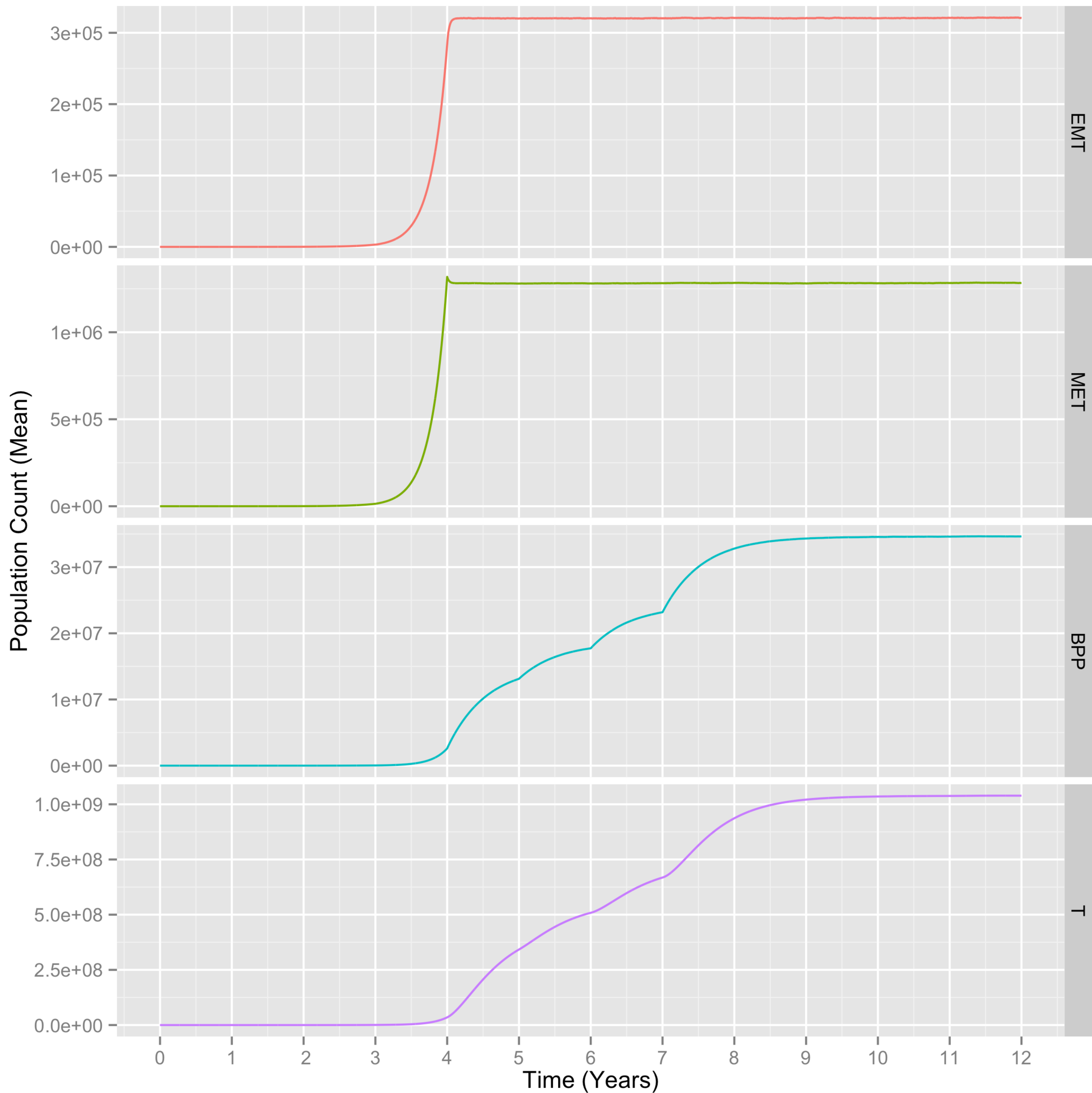

Supplement: S1 Appendix — shows the predicted population trajectories for each cell species, including breast cancer stem cells (BCSCs), bipotent progenitors (BPP) and terminally differentiated cells (TCs) corresponding to the simulation results for total cell counts presented in Fig 2. (PDF) [file pone.0135797.s003.pdf]
