# Supplementary material for: Modeling of Cancer Stem Cell State Transitions Predicts Therapeutic Response
Source: PLoS One. 2015 Sep 23;10(9):e0135797. doi: 10.1371/journal.pone.0135797 (PMC4580445; doi:10.1371/journal.pone.0135797)
Supplement: S2 Appendix — (PDF) [file pone.0135797.s004.pdf]

```xml
<?xml version='1.0' encoding='UTF-8' standalone='no'?>
<sbml xmlns="http://www.sbml.org/sbml/level2/version4" level="2" version="4">
  <model id="model" name="BCSC Model">
    <listOfCompartments>
      <compartment id="compartment_0000001" name="compartment" size="1.0"/>
    </listOfCompartments>
    <listOfSpecies>
      <species id="species_0000001" initialAmount="5" name="EMT"
compartment="compartment_0000001"/>
      <species id="species_0000002" initialAmount="20" name="MET"
compartment="compartment_0000001"/>
      <species id="species_0000003" initialAmount="100" name="BPP"
compartment="compartment_0000001"/>
      <species id="species_0000004" initialAmount="500" name="T"
compartment="compartment_0000001"/>
    </listOfSpecies>
    <listOfParameters>
      <parameter id="parameter_0000001" constant="true" name="EMTrate" value="0.08"/>
      <parameter id="parameter_0000002" constant="true" name="METrate" value="0.02"/>
      <parameter id="parameter_0000003" constant="false" name="BCSCsymsrate" value="0.003375"/>
      <parameter id="parameter_0000004" constant="false" name="BCSCasymsrate" value="0.027"/>
      <parameter id="parameter_0000005" constant="false" name="BCSCsymdrate" value="0.003375"/>
      <parameter id="parameter_0000006" constant="true" name="BPPsymrate" value="0.31"/>
      <parameter id="parameter_0000007" constant="true" name="BPPdeathrate" value="0.015"/>
      <parameter id="parameter_0000008" constant="true" name="BPPdiffrate" value="0.3"/>
      <parameter id="parameter_0000009" constant="true" name="Tdeathrate" value="0.01"/>
    </listOfParameters>
    <listOfReactions>
      <reaction id="reaction_0000001" name="Epithelial-Mesenchymal">
        <listOfReactants>
          <speciesReference species="species_0000001" stoichiometry="1"/>
        </listOfReactants>
        <listOfProducts>
          <speciesReference species="species_0000002" stoichiometry="1"/>
        </listOfProducts>
        <kineticLaw>
          <math xmlns="http://www.w3.org/1998/Math/MathML">
            <apply>
              <times/>
              <ci> parameter_0000001 </ci>
              <ci> species_0000001 </ci>
            </apply>
          </math>
        </kineticLaw>
      </reaction>
      <reaction id="reaction_0000002" name="Mesenchymal-Epithelial">
        <listOfReactants>
          <speciesReference species="species_0000002" stoichiometry="1"/>
        </listOfReactants>
        <listOfProducts>
          <speciesReference species="species_0000001" stoichiometry="1"/>
        </listOfProducts>
        <kineticLaw>
          <math xmlns="http://www.w3.org/1998/Math/MathML">
            <apply>
              <times/>
              <ci> parameter_0000002 </ci>
              <ci> species_0000002 </ci>
            </apply>
          </math>
        </kineticLaw>
      </reaction>
      <reaction id="reaction_0000003" name="Symmetric Self-Renewal (BCSC)">
        <listOfReactants>
          <speciesReference species="species_0000002" stoichiometry="2"/>
        </listOfReactants>
        <listOfProducts>
          <speciesReference species="species_0000002" stoichiometry="2"/>
```

```
    &lt;/listOfProducts&gt;
    &lt;kineticLaw&gt;
      &lt;math xmlns="http://www.w3.org/1998/Math/MathML"&gt;
        &lt;apply&gt;
          &lt;times/&gt;
          &lt;ci&gt; parameter_0000003 &lt;/ci&gt;
          &lt;ci&gt; species_0000002 &lt;/ci&gt;
        &lt;/apply&gt;
      &lt;/math&gt;
    &lt;/kineticLaw&gt;
  &lt;/reaction&gt;
  &lt;reaction id="reaction_0000004" name="Asymmetric Self-Renewal (BCSC)"&gt;
    &lt;listOfReactants&gt;
      &lt;speciesReference species="species_0000002" stoichiometry="1"/&gt;
    &lt;/listOfReactants&gt;
    &lt;listOfProducts&gt;
      &lt;speciesReference species="species_0000002" stoichiometry="1"/&gt;
      &lt;speciesReference species="species_0000003" stoichiometry="1"/&gt;
    &lt;/listOfProducts&gt;
    &lt;kineticLaw&gt;
      &lt;math xmlns="http://www.w3.org/1998/Math/MathML"&gt;
        &lt;apply&gt;
          &lt;times/&gt;
          &lt;ci&gt; parameter_0000004 &lt;/ci&gt;
          &lt;ci&gt; species_0000002 &lt;/ci&gt;
        &lt;/apply&gt;
      &lt;/math&gt;
    &lt;/kineticLaw&gt;
  &lt;/reaction&gt;
  &lt;reaction id="reaction_0000005" name="Symmetric Differentiation (BCSC)"&gt;
    &lt;listOfReactants&gt;
      &lt;speciesReference species="species_0000002" stoichiometry="1"/&gt;
    &lt;/listOfReactants&gt;
    &lt;listOfProducts&gt;
      &lt;speciesReference species="species_0000003" stoichiometry="2"/&gt;
    &lt;/listOfProducts&gt;
    &lt;kineticLaw&gt;
      &lt;math xmlns="http://www.w3.org/1998/Math/MathML"&gt;
        &lt;apply&gt;
          &lt;times/&gt;
          &lt;ci&gt; parameter_0000005 &lt;/ci&gt;
          &lt;ci&gt; species_0000002 &lt;/ci&gt;
        &lt;/apply&gt;
      &lt;/math&gt;
    &lt;/kineticLaw&gt;
  &lt;/reaction&gt;
  &lt;reaction id="reaction_0000006" name="Symmetric Self-Renewal (BPP)"&gt;
    &lt;listOfReactants&gt;
      &lt;speciesReference species="species_0000003" stoichiometry="1"/&gt;
    &lt;/listOfReactants&gt;
    &lt;listOfProducts&gt;
      &lt;speciesReference species="species_0000003" stoichiometry="2"/&gt;
    &lt;/listOfProducts&gt;
    &lt;kineticLaw&gt;
      &lt;math xmlns="http://www.w3.org/1998/Math/MathML"&gt;
        &lt;apply&gt;
          &lt;times/&gt;
          &lt;ci&gt; parameter_0000006 &lt;/ci&gt;
          &lt;ci&gt; species_0000003 &lt;/ci&gt;
        &lt;/apply&gt;
      &lt;/math&gt;
    &lt;/kineticLaw&gt;
  &lt;/reaction&gt;
  &lt;reaction id="reaction_0000007" name="Death (BPP)"&gt;
    &lt;listOfReactants&gt;
      &lt;speciesReference species="species_0000003" stoichiometry="1"/&gt;
    &lt;/listOfReactants&gt;
    &lt;kineticLaw&gt;
```

```
          <math xmlns="http://www.w3.org/1998/Math/MathML">
            <apply>
              <times/>
              <ci> parameter_0000007 </ci>
              <ci> species_0000003 </ci>
            </apply>
          </math>
        </kineticLaw>
      </reaction>
      <reaction id="reaction_0000008" name="Differentiation (BPP)">
        <listOfReactants>
          <speciesReference species="species_0000003" stoichiometry="1"/>
        </listOfReactants>
        <listOfProducts>
          <speciesReference species="species_0000004" stoichiometry="1"/>
        </listOfProducts>
        <kineticLaw>
          <math xmlns="http://www.w3.org/1998/Math/MathML">
            <apply>
              <times/>
              <ci> parameter_0000008 </ci>
              <ci> species_0000003 </ci>
            </apply>
          </math>
        </kineticLaw>
      </reaction>
      <reaction id="reaction_0000009" name="Death (T)">
        <listOfReactants>
          <speciesReference species="species_0000004" stoichiometry="1"/>
        </listOfReactants>
        <kineticLaw>
          <math xmlns="http://www.w3.org/1998/Math/MathML">
            <apply>
              <times/>
              <ci> parameter_0000009 </ci>
              <ci> species_0000004 </ci>
            </apply>
          </math>
        </kineticLaw>
      </reaction>
    </listOfReactions>
    <listOfEvents>
      <event id="event_0000001" name="year1change">
        <trigger>
          <math xmlns="http://www.w3.org/1998/Math/MathML">
            <apply>
              <geq/>
              <csymbol encoding="text" definitionURL="http://www.sbml.org/sbml/symbols/time"> t
</csymbol>
              <cn type="integer"> 365 </cn>
            </apply>
          </math>
        </trigger>
        <listOfEventAssignments>
          <eventAssignment variable="parameter_0000003">
            <math xmlns="http://www.w3.org/1998/Math/MathML">
              <cn> 0.005785714 </cn>
            </math>
          </eventAssignment>
          <eventAssignment variable="parameter_0000005">
            <math xmlns="http://www.w3.org/1998/Math/MathML">
              <cn> 0.005785714 </cn>
            </math>
          </eventAssignment>
        </listOfEventAssignments>
      </event>
      <event id="event_0000002" name="year2change">
        <trigger>
```

```
          <math xmlns="http://www.w3.org/1998/Math/MathML">
            <apply>
              <geq/>
              <csymbol encoding="text" definitionURL="http://www.sbml.org/sbml/symbols/time"> t 
</csymbol>
              <cn type="integer"> 730 </cn>
            </apply>
          </math>
        </trigger>
        <listOfEventAssignments>
          <eventAssignment variable="parameter_0000003">
            <math xmlns="http://www.w3.org/1998/Math/MathML">
              <cn> 0.009 </cn>
            </math>
          </eventAssignment>
          <eventAssignment variable="parameter_0000005">
            <math xmlns="http://www.w3.org/1998/Math/MathML">
              <cn> 0.009 </cn>
            </math>
          </eventAssignment>
        </listOfEventAssignments>
      </event>
      <event id="event_0000003" name="year3change">
        <trigger>
          <math xmlns="http://www.w3.org/1998/Math/MathML">
            <apply>
              <geq/>
              <csymbol encoding="text" definitionURL="http://www.sbml.org/sbml/symbols/time"> t 
</csymbol>
              <cn type="integer"> 1095 </cn>
            </apply>
          </math>
        </trigger>
        <listOfEventAssignments>
          <eventAssignment variable="parameter_0000003">
            <math xmlns="http://www.w3.org/1998/Math/MathML">
              <cn> 0.0135 </cn>
            </math>
          </eventAssignment>
          <eventAssignment variable="parameter_0000005">
            <math xmlns="http://www.w3.org/1998/Math/MathML">
              <cn> 0.0135 </cn>
            </math>
          </eventAssignment>
        </listOfEventAssignments>
      </event>
      <event id="event_0000004" name="year4change">
        <trigger>
          <math xmlns="http://www.w3.org/1998/Math/MathML">
            <apply>
              <geq/>
              <csymbol encoding="text" definitionURL="http://www.sbml.org/sbml/symbols/time"> t 
</csymbol>
              <cn type="integer"> 1460 </cn>
            </apply>
          </math>
        </trigger>
        <listOfEventAssignments>
          <eventAssignment variable="parameter_0000003">
            <math xmlns="http://www.w3.org/1998/Math/MathML">
              <cn> 0.02025 </cn>
            </math>
          </eventAssignment>
          <eventAssignment variable="parameter_0000005">
            <math xmlns="http://www.w3.org/1998/Math/MathML">
              <cn> 0.02025 </cn>
            </math>
          </eventAssignment>
```

```
        </listOfEventAssignments>
      </event>
      <event id="event_0000005" name="year5change">
        <trigger>
          <math xmlns="http://www.w3.org/1998/Math/MathML">
            <apply>
              <geq/>
              <csymbol encoding="text" definitionURL="http://www.sbml.org/sbml/symbols/time"> t
</csymbol>
              <cn type="integer"> 1825 </cn>
            </apply>
          </math>
        </trigger>
        <listOfEventAssignments>
          <eventAssignment variable="parameter_0000003">
            <math xmlns="http://www.w3.org/1998/Math/MathML">
              <cn> 0.0315 </cn>
            </math>
          </eventAssignment>
          <eventAssignment variable="parameter_0000005">
            <math xmlns="http://www.w3.org/1998/Math/MathML">
              <cn> 0.0315 </cn>
            </math>
          </eventAssignment>
        </listOfEventAssignments>
      </event>
      <event id="event_0000006" name="year6change">
        <trigger>
          <math xmlns="http://www.w3.org/1998/Math/MathML">
            <apply>
              <geq/>
              <csymbol encoding="text" definitionURL="http://www.sbml.org/sbml/symbols/time"> t
</csymbol>
              <cn type="integer"> 2190 </cn>
            </apply>
          </math>
        </trigger>
        <listOfEventAssignments>
          <eventAssignment variable="parameter_0000003">
            <math xmlns="http://www.w3.org/1998/Math/MathML">
              <cn> 0.054 </cn>
            </math>
          </eventAssignment>
          <eventAssignment variable="parameter_0000005">
            <math xmlns="http://www.w3.org/1998/Math/MathML">
              <cn> 0.054 </cn>
            </math>
          </eventAssignment>
        </listOfEventAssignments>
      </event>
      <event id="event_0000007" name="year7change">
        <trigger>
          <math xmlns="http://www.w3.org/1998/Math/MathML">
            <apply>
              <geq/>
              <csymbol encoding="text" definitionURL="http://www.sbml.org/sbml/symbols/time"> t
</csymbol>
              <cn type="integer"> 2555 </cn>
            </apply>
          </math>
        </trigger>
        <listOfEventAssignments>
          <eventAssignment variable="parameter_0000003">
            <math xmlns="http://www.w3.org/1998/Math/MathML">
              <cn> 0.054 </cn>
            </math>
          </eventAssignment>
          <eventAssignment variable="parameter_0000005">
```

```
            <math xmlns="http://www.w3.org/1998/Math/MathML">
              <cn> 0.054 </cn>
            </math>
          </eventAssignment>
        </listOfEventAssignments>
      </event>
      <event id="event_0000008" name="year8change">
        <trigger>
          <math xmlns="http://www.w3.org/1998/Math/MathML">
            <apply>
              <geq/>
              <csymbol encoding="text" definitionURL="http://www.sbml.org/sbml/symbols/time"> t
</csymbol>
              <cn type="integer"> 2920 </cn>
            </apply>
          </math>
        </trigger>
        <listOfEventAssignments>
          <eventAssignment variable="parameter_0000003">
            <math xmlns="http://www.w3.org/1998/Math/MathML">
              <cn> 0.054 </cn>
            </math>
          </eventAssignment>
          <eventAssignment variable="parameter_0000005">
            <math xmlns="http://www.w3.org/1998/Math/MathML">
              <cn> 0.054 </cn>
            </math>
          </eventAssignment>
        </listOfEventAssignments>
      </event>
    </listOfEvents>
  </model>
</sbml>
```
